# Mechanisms Mediating Tart Cherry and Fish Oil Metabolic Effects in Diet-Induced (C57BL/6J) and Genetically (TALYHO/Jng) Obese Mice

**DOI:** 10.3390/nu16234179

**Published:** 2024-12-01

**Authors:** Maryam Seifishahpar, Jung Han Kim, Jacaline K. Parkman, Ana Rhode, Kalhara Menikdiwela, Yujiao Zu, Shane Scoggin, Logan Freeman, Nishan Sudheera Kalupahana, Naima Moustaid-Moussa

**Affiliations:** 1 Department of Nutritional Sciences, Texas Tech University, Lubbock, TX 79409, USA; mseifish@ttu.edu (M.S.);; 2 Obesity Research Institute, Texas Tech University, Lubbock, TX 79409, USA; 3 Department of Biomedical Sciences, School of Medicine, Marshall University, Huntington, WV 25755, USA; kimj@marshall.edu (J.H.K.);; 4 Department of Nutrition and Health, College of Medicine and Health Sciences, United Arab Emirates University, Al Ain P.O. Box 15551, United Arab Emirates; nkalupahana@uaeu.ac.ae; 5 Institute for One Health Innovation, Texas Tech University, Lubbock, TX 79409, USA; 6 Texas Tech Health Sciences Center, Lubbock, TX 79430, USA

**Keywords:** obesity, inflammation, tart cherry, fish oil, white adipose tissue

## Abstract

Background/Objectives: Obesity is a major public health concern that increases the risk of chronic diseases. In obesity, adipose tissue undergoes remodeling, which is associated with chronic low-grade inflammation and disruption of its homeostatic mechanisms including endoplasmic reticulum (ER) function and autophagy. Fish oil (FO) and tart cherry (TC) have known anti-inflammatory properties. We hypothesized that while TC and FO individually decrease inflammation, their combined effects will be greater and will be either synergistic or additive in regulating inflammation and other adipose tissue functions. Methods: Here, we conducted gene expression analyses, using qRT-PCR, on gonadal white adipose tissues from a previous study where male and female C57BL/6J (B6) and TALLYHO/Jng (TH) mice were fed low fat (LF), high fat (HF), or HF diets supplemented with TC, FO, or TC + FO for 14 weeks from weaning. Data was statistically analyzed by one or two-way ANOVA, using GraphPad Prism. Results: HF diet increased adiposity and upregulated markers of inflammation, ER stress, and autophagy compared to the LF diet in both mouse models. While both TC and FO supplementation individually reduced the expression of inflammatory, ER stress, and autophagy markers on HF diet, their combination showed no consistent additive or synergistic effects. Conclusions: Overall, our findings suggest that although TC and FO effectively mitigate inflammation in white adipose tissue, their combined use did not result in synergistic or additive effects of the two interventions.

## 1. Introduction

Obesity is a chronic progressive disease associated with an elevated risk of comorbid conditions like diabetes, cardiovascular diseases, and several cancers, contributing to increased rates of morbidity and mortality [1,2]. Obesity is attributed to genetic, environmental, and lifestyle factors [3,4]. The pathogenesis of obesity involves prolonged periods of positive energy balance, leading to the accumulation of excess energy in the form of triglycerides within the white adipose tissue (WAT) [5,6]. Excessive adiposity triggers WAT macrophage infiltration, disrupting the balance between the secretion of pro-inflammatory and anti-inflammatory mediators, resulting in a state of chronic low-grade inflammation, which further exacerbates metabolic derangements [7,8]. Moreover, excessive lipid accumulation and adipocyte hypertrophy lead to endoplasmic reticulum (ER) stress. This stress arises from an imbalance between the increased demand for protein folding and the limited folding capacity of ER. In response to ER stress, cells activate a complex adaptive signaling network known as the unfolded protein response (UPR) [9]. On the one hand, if the UPR fails to restore ER homeostasis, prolonged ER stress can trigger apoptosis, thereby contributing to cellular dysfunction, inflammation, and metabolic complications. On the other hand, to mitigate ER stress and restore homeostasis, cells also activate autophagy, a process that degrades misfolded/unfolded proteins and damaged organelles. Therefore, in the context of obesity, sustained ER stress, dysregulated UPR signaling, and impaired autophagy collectively contribute to the development of insulin resistance, chronic low-grade inflammation, and metabolic complications [10].

Management of obesity includes lifestyle and behavioral changes, pharmacotherapy, and bariatric surgery [11]. Dietary interventions using bioactive compounds with antioxidant and anti-inflammatory properties have emerged as a promising therapeutic approach to mitigate obesity and associated metabolic conditions [12]. These compounds include long-chain omega-3 polyunsaturated fatty acids (omega-3 PUFAs) from fish oil (FO), anthocyanins from cherries and berries, curcumin, and others [13,14].

Tart cherries (TC) are known for their high content of anthocyanins, compared to other vegetables and fruits [15,16]. Extensive in vitro and in vivo studies underscore the health-promoting effects of anthocyanins on improving lipid metabolism by reducing fatty acid synthesis, increasing fatty acid oxidation, as well as reducing oxidative stress and inflammation [17,18]. Our previous research demonstrated that TC extract effectively reduced obesity-associated inflammation in genetically obese Zucker rats, by targeting the nuclear factor kappa B (NF-κB) pathway, as evidenced in both cell and animal studies [8]. This inhibition was associated with reduced levels of pro-inflammatory cytokines, including interleukin 6 (Il6), tumor necrosis factor-alpha (Tnfα), and interleukin 1beta (Il1β), underscoring the potential of TC extract as a therapeutic intervention for mitigating obesity-induced inflammation [8].

Another compound of interest is FO, which is a major source of anti-inflammatory long-chain omega-3 PUFAs, namely eicosapentaenoic acid (EPA) and docosahexaenoic acid (DHA) [19,20]. The beneficial metabolic effects of omega-3 PUFAs are partially attributed to reduced inflammation and insulin resistance [21]. Indeed, EPA supplementation effectively mitigated diet-induced obesity in male C57BL/6J (B6) mice, which was accompanied by attenuated systemic and adipose tissue inflammation [13]. The underlying mechanisms for these beneficial effects of omega-3 PUFAs included upregulation of adipose tissue and circulating adiponectin levels and downregulation of circulating pro-inflammatory cytokines concomitant with reduced macrophage infiltration, reactive oxygen species, lipid accumulation, and adipocyte size in WAT [13,22].

While most previous studies have investigated the individual effects of TC or FO on inflammation and metabolism, to our knowledge, no study has looked at the combined beneficial effects of these two bioactives. We hypothesized that TC and FO work through different mechanisms to alleviate obesity-associated inflammation, and hence would exert synergistic effects. In our previous study, we tested this hypothesis and showed that while TC, FO, or the combination of TC and FO were not able to prevent HF diet-induced obesity in B6 and genetically obese TALLYHO/Jng (TH) mice [23,24,25], FO was able to attenuate glucose intolerance in TH males [26]. Further, both TC and FO decreased plasma inflammatory markers [26]. TH mice are an inbred polygenic model of human obesity and type 2 diabetes, with disease susceptibility influenced by multiple genetic polymorphisms [23,24]. Compared to B6 mice, TH mice are more susceptible to hyperglycemia, hyperlipidemia, and weight gain when fed a diet high in saturated fat and sucrose [25]. In the current study, we further investigated the mechanisms mediating these effects of TC and FO in B6 and TH mice. Specifically, we determined whether the systemic metabolic and anti-inflammatory effects of these compounds are due to their effects on adipose tissue. We hypothesize that TC and FO reduce systemic inflammatory markers in B6 and TH via the modulation of gene expression in WAT. We tested markers of inflammation, ER stress, autophagy, and lipid oxidation in adipose tissue.

## 2. Materials and Methods

### 2.1. Animal Study and Diets

**Figure 1:**
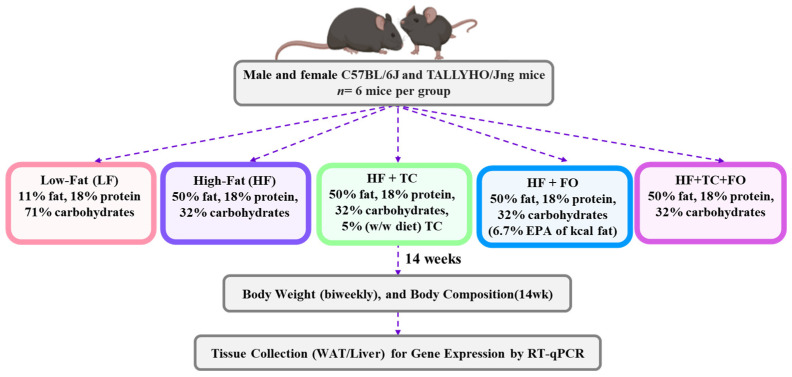
Study design.

The WAT used in this study were from a previously reported animal experiment [26]. Briefly, male and female TH mice originating from our breeding colony [23], and B6 mice purchased from the Jackson Laboratory (Bar Harbor, ME, USA), were used. Commencing at 4 weeks of age, both male and female B6 and TH mice were fed five distinct diets, namely low fat (LF), high fat (HF), and HF supplemented with tart cherry (TC), fish oil (FO), or a combination of TC and FO (TC + FO) (sourced from Research Diets, New Brunswick, NJ, USA) (*n* = 6 mice per group). Throughout the study, the mice were maintained on their assigned diets, provided in pellet form. The freeze-dried TC powder utilized in our study was a generous contribution from the Cherry Marketing Institute, (Dewitt, MI, USA). This TC powder was from Montmorency tart cherries, which were individually quickly frozen and processed into a powder form by Van Drunen Farms (Momence, IL, USA). The menhaden oil utilized for FO supplementation comprised 32.18% omega-3 PUFAs, with EPA and DHA constituting 13.52% and 9.11%, respectively (sourced from Omega Protein, Reedville, VA, USA). The detailed diet composition is in Table 1, and the study design is presented in Figure 1. After 14 weeks of intervention, the mice were euthanized, and the WAT was harvested and stored at −80 °C for further analyses.

Mice were provided with free access to food and water during the study. The mice were housed in cages with controlled humidity and temperature and were exposed to a 12-h cycle of light and darkness. At the end of the dietary intervention, the mice were euthanized through CO_2_ asphyxiation followed by exsanguination via cardiac puncture. Gonadal (epididymal or parametrial) fat pads were dissected, rapidly frozen in liquid nitrogen, and stored at −80 °C for further analysis. All animal protocols were approved by the Marshall University Animal Care and Use Committee (IACUC) (protocol #507, approved on 9 May 2018) and Texas Tech University IACUC (protocol #19034-04, approved on 8 April 2019).

### 2.2. Body Composition and Adiposity Index

At the age of 14 weeks, fat mass and lean mass of the mice were determined through quantitative magnetic resonance imaging using an EchoMRI-100 whole body composition analyzer (Echo Medical Systems, Houston, TX, USA). Data were collected by taking the median of five measurements for each animal. To calculate the adiposity index, the following formula was used: Adiposity index (%) = (fat mass (g)/total body weight (g)) × 100 [27].

### 2.3. RNA Extraction, cDNA Preparation, and Real-Time PCR

Total RNA was extracted from WAT (Gonadal fat pads) using the Quick RNA mini-prep kit (ZYMO Research, Irvine, CA, USA). Subsequently, cDNA was synthesized through reverse transcription using Maxima reverse transcriptase. To measure gene expression levels, a real-time quantitative polymerase chain reaction (RT-qPCR) was performed using SYBR green master mix (Thermo Fisher Scientific, Waltham, MA, USA). Gene expression levels of individual genes were normalized to that of housekeeping genes, glyceraldehyde 3-phosphate dehydrogenase (GAPDH), or 18S ribosomal RNA in the same sample. The sequence of primers used in the RT-qPCR analysis is listed in Table 2.

### 2.4. Statistical Analyses

Statistical analysis was conducted for each strain using GraphPad Prism version 8.0.2. A two-way ANOVA evaluated the main effects of diet (HF or LF) and sex (male or female), as well as their interaction, on the dependent variables, followed by the multiple comparison post-test with Tukey correction. Subsequently, a one-way ANOVA was performed to assess the main effects of supplemented diets (TC, FO, or TC + FO), followed by Tukey’s post-test for multiple comparisons. Additionally, to determine the presence of a sex effect, two-way ANOVA was again performed including a main effect for sex (male and female), and supplemented diet (TC or FO or TC + FO). Significant findings were further explored with Tukey-corrected post hoc comparisons. Results are presented as mean ± SEM (standard error of the mean), with statistical significance considered at *p* < 0.05.

## 3. Results

For each experiment, data are presented for LF vs. HF comparison followed by HF vs. supplementation with TC, FO, or TC + FO.

### 3.1. Effects of HF Diet on Adiposity

**Figure 2:**
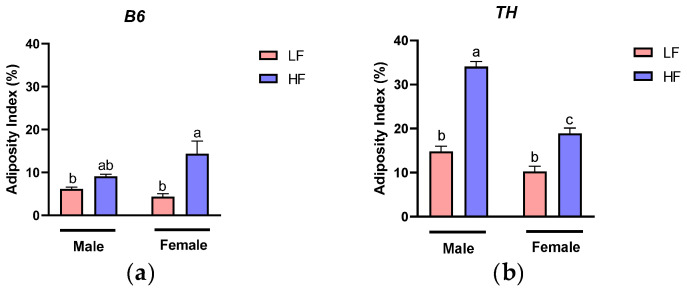
Adiposity index. (**a**) Male and female C57BL/6J (B6), (**b**) male and female TALLYHO/Jng (TH) mice. Data are expressed as mean ± SEM. LF, low fat, HF, high fat. Different letters indicate significant differences between groups. *p* < 0.05; *n* = 6 mice per group.

Diets rich in fat have consistently been linked to increases in body weight and fat mass across various rodent models [28,29,30]. Accordingly, we first examined the effects of HF diets on obesity in male and female B6 and TH mice. Our findings revealed that female B6 mice on the HF diet exhibited a modest increase in the adiposity index compared to ones on the LF diet (10% increase; *p* = 0.0026), while in male B6 mice there was no difference between the HF and LF diets (*p* = 0.72) (Figure 2a). In contrast, both TH male and female mice on the HF diet had significantly higher adiposity indices compared to those on the LF diet (*p* < 0.0001, *p* = 0.0004, respectively) (Figure 2b). These findings are consistent with our previously reported data, indicating that HF diets lead to increased body weight and fat mass in both B6 and TH mice, with TH mice exhibiting a more pronounced obesogenic response compared to B6 mice [26].

### 3.2. Effects of Supplemental TC, FO, and Their Combination on Adiposity

**Figure 3:**
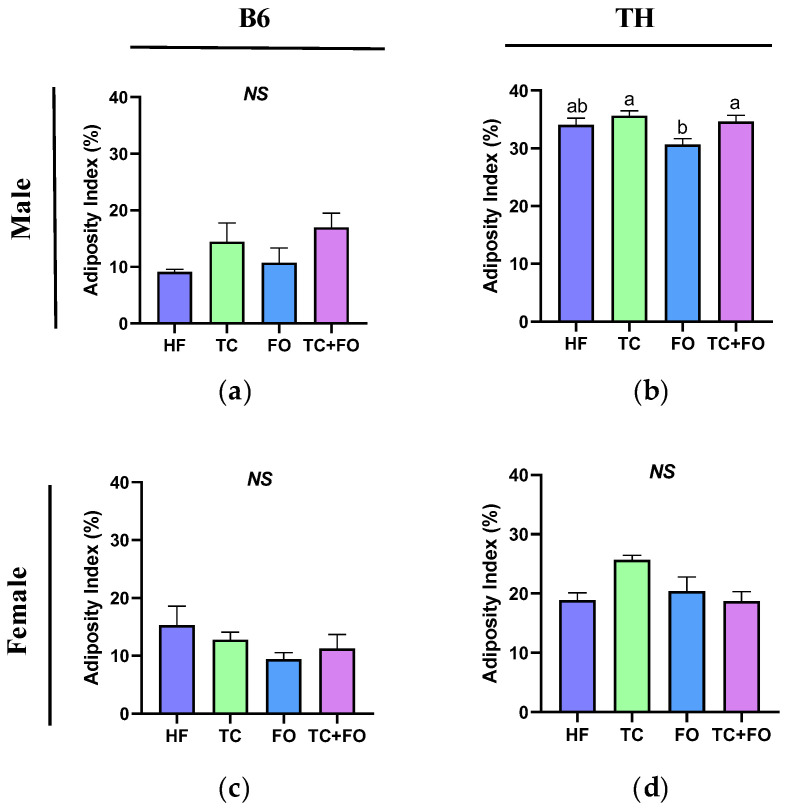
Adiposity index. (**a**,**c**) Male and female C57BL/6J (B6), (**b**,**d**) male and female TALLYHO/Jng (TH) mice. Data are expressed as mean ± SEM. HF, high fat (HF) diets and HF diets supplemented with tart cherry (TC), fish oil (FO), and their combination (TC + FO). Different letters indicate significant differences between groups. *p* < 0.05; *n* = 6 mice per group.

To evaluate the anti-obesity effects of supplemental TC, FO, and their combination, we next measured the adiposity index of both B6 and TH mice fed HF diets without and with supplantation. Our findings demonstrate that TC, FO, or their combination have limited effects in reducing diet-induced obesity in both B6 and TH models (Figure 3a–d), consistent with our previous observations on body weight or fat mass [26].

### 3.3. Effects of HF Diet on Inflammatory Markers in WAT

Obesity is closely associated with increased WAT inflammation, contributing to metabolic dysregulation. Hence, we assessed the mRNA expression levels of key inflammatory genes, including *Il6*, *Tnfα*, *Mcp1*, and *Tlr4*.

In B6 mice, two-way ANOVA revealed significant main effects of sex and diet on the expression of *Il6* (F (1, 18) = 17.59, *p* = 0.0005, F (1, 18) = 7.388, *p* = 0.0141). The main effects of diet were also significant for *Tnfα* and *Tlr4* (F (1, 17) = 6.702, *p*= 0.0191, F (1, 15) = 39.04, *p* < 0.0001). Interactions between sex and diet were observed in the expression of *Il6* (F (1, 18) = 7.489, *p* = 0.0136), and *Tnfα* (F (1, 17) = 5.341, *p* = 0.0336) (Table 3).

In TH mice, two-way ANOVA confirmed significant main effects of both sex and diet on *Il6* (F (1, 19) = 16.28, *p* = 0.0007, F (1, 19) = 59.38, *p* < 0.0001), *Mcp1* (F (1, 20) = 7.770, *p* = 0.0114, F (1, 20) = 9.775, *p* = 0.0053) and *Tlr4* (F (1, 20) = 16.14, *p* = 0.0007, F (1, 20) = 8.244, *p* = 0.0094). No interactions between sex and diet were detected (Table 4).

**Figure 4:**
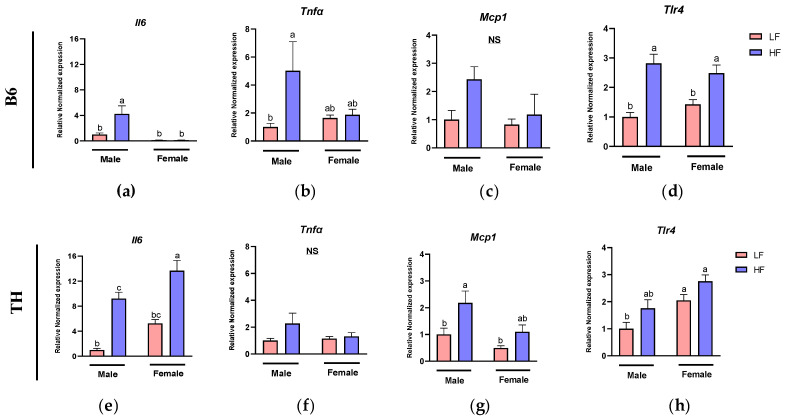
Expression of inflammation-related genes in white adipose tissue (WAT). WAT inflammatory marker genes include (**a**,**e**) interleukin 6 (*Il6*), (**b**,**f**) tumor necrosis factor alpha (*Tnfα*), (**c**,**g**) monocyte chemoattractant protein1 (*Mcp1*), and (**d**,**h**) toll-like receptor 4 (*Tlr4*). Data are expressed as mean ± SEM. LF, low fat, HF, high fat. Different letters indicate significant differences between groups. *p* < 0.05; *n* = 6 mice per group.

In B6 mice, males exhibited significantly increased WAT expression levels of *Il6* (*p* = 0.0058), *Tnfα* (*p* = 0.0177), and *Tlr4* (*p* = 0.0006) in response to the HF diet compared to the LF diet (Figure 4a–d). However, in females, we only observed a higher expression of *Tlr4* (*p* = 0.0143) in the HF group compared to the LF group (Figure 4d), suggesting that males have higher levels of WAT inflammation than females when challenged with the HF diet. Similar to B6, TH male mice in the HF group showed a significant increase in the expression levels of *Il6* (*p* = 0.0003) and *Mcp1* (*p* = 0.0378) compared to those fed with the LF diet (Figure 4e,g). In TH female mice, HF diet feeding resulted in a significant increase in *Il6* (*p* = 0.0001) expression compared to the LF diet (Figure 4e). Although there was an upward trend in the expression of *Tnfα*, *Mcp1*, and *Tlr4* (Figure 4f–h) in HF-fed females, these changes were not significant.

Considering the imbalance between pro-inflammatory M1 and anti-inflammatory M2 macrophage polarization in obese WAT, which plays a pivotal role in obesity-related chronic inflammation, we next measured the expression of M1 and M2 macrophage markers in WAT.

In B6 mice, two-way ANOVA revealed significant main effects of diet on the expression of *Il1β* (F (1, 17) = 9.251, *p* = 0.0074) and *Cd80* (F (1, 18) = 13.23, *p* = 0.0019). Additionally, a significant main effect of sex was observed for *Cd80* (F (1, 18) = 4.796, *p* = 0.0419), indicating differing levels of expression between sexes. Also, *Arg1* showed pronounced main effects of both diet (F (1, 16) = 43.20, *p* < 0.0001) and sex (F (1, 16) = 23.74, *p* = 0.0002). Furthermore, a significant interaction between sex and diet was observed for *Arg1* (F (1, 16) = 6.978, *p* = 0.0178), suggesting that the dietary response varies significantly between male and female mice (Table 3).

Two-way ANOVA results for TH mice highlighted significant main effects of sex and diet on *Il1β* (F (1, 18) = 39.10, *p* < 0.0001, F (1, 18) = 17.23, *p* = 0.0006). Similarly, *Arg1* also showed significant main effects of sex (F (1, 20) = 11.13, *p* = 0.0033) and diet (F (1, 20) = 35.53, *p* < 0.0001). However, for *Cd80*, only diet demonstrated a significant main effect (F (1, 20) = 40.65, *p* = <0.0001). There were also no significant interactions between diet and sex for these genes (Table 4).

**Figure 5:**
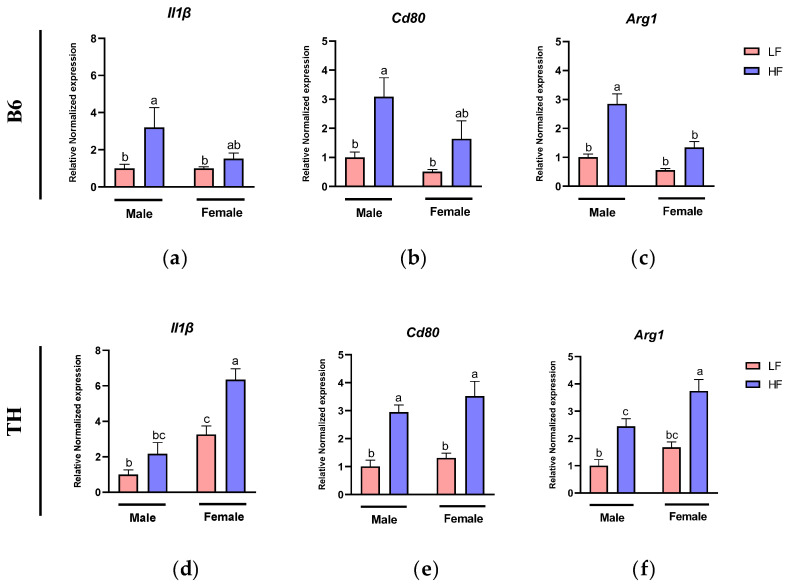
Expression of macrophage marker genes in white adipose tissue (WAT). M1 type markers include (**a**,**d**) *Il1β*, (**b**,**e**) *Cd80*, M2 type markers include (**c**,**f**) Arginase 1 (*Arg1*). Data are expressed as mean ± SEM. LF, low fat, HF, high fat. Different letters indicate significant differences between groups. *p* < 0.05; *n* = 6 mice per group.

We observed that, in B6 male mice, the HF diet significantly increased the expression levels of genes involved in M1 macrophages, including *Il1β* (*p* = 0.0177) and *Cd80* (*p* = 0.0113), compared to the LF diet (Figure 5a,b). Similar trends were observed in B6 females, but no significant differences were detected (Figure 5a,b). Unexpectedly, we found that the expression level of *Arg1*, an M2 macrophage gene, was also upregulated in the B6 males fed with the HF diet (*p* < 0.0001). A similar result was observed in females, but no significant difference was detected (Figure 5c).

In TH male mice, the HF diet led to a significant increase in M1 (*Cd80*, *p* = 0.0022) and M2 (*Arg1*, *p* = 0.0120) markers compared to the LF diet (Figure 5e,f). A similar upward trend was observed for *Il1β* in TH male mice, although it was not significant (Figure 5d). Likewise, TH female mice on an HF diet demonstrated a significant increase in both M1 (*Il1β*, *p* = 0.0022; *Cd80*, *p* = 0.0022) and M2 (*Arg1*, *p* = 0.0004) markers compared to their LF diet counterparts (Figure 5d–f).

### 3.4. Effects of Supplemental TC, FO, and Their Combination on Inflammatory Markers in WAT

Next, we evaluated the anti-inflammatory effects of TC, FO, and their combination in the WAT of B6 and TH mice fed the HF diet by measuring the gene expression of key inflammatory markers such as *Mcp1*, *Tlr4*, and *Il1β*.

In B6 mice, two-way ANOVA indicated a significant main effect of sex on the expression of *Tlr4* (F (1, 30) = 34.97, *p* < 0.0001). Additionally, significant interactions between diet and sex were observed for *Tlr4* (F (3, 30) = 8.887, *p* = 0.0002) (Table 5).

In TH mice, two-way ANOVA indicated significant main effects of both sex and diet on the expression of *Mcp1* (F (1, 38) = 11.11, *p* = 0.0019; F (3, 38) = 2.988, *p* = 0.0430) and *Tlr4* (F (1, 38) = 7.640, *p* = 0.0088; F (3, 38) = 4.721, *p* = 0.0068). A significant main effect of sex was also observed for *Il1β* (F (1, 35) = 15.76, *p* = 0.0003). There were no significant interactions between diet and sex for these genes (Table 6).

**Figure 6:**
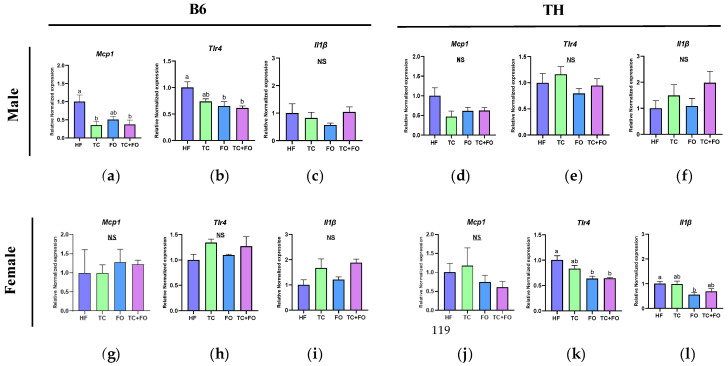
Gene expression levels of (**a**,**d**,**g**,**j**) *Mcp1*, (**b**,**e**,**h**,**k**) *Tlr4*, (**c**,**f**,**i**,**l**) *Il1β* in WAT of mice fed high fat (HF) diets and HF diets supplemented with tart cherry (TC), fish oil (FO), and their combination (TC + FO). Group means labeled with different letters are significantly different *p* < 0.05; *n* = 6 mice per group.

In B6 male mice, compared to the HF diet, the consumption of the TC and FO combination led to a downregulation of *Mcp1* (*p* = 0.02) and *Tlr4* (*p* = 0.0145) gene expression (Figure 6a,b). Individually, TC supplementation was found to reduce *Mcp1* (*p* = 0.012) expression (Figure 6a), and FO supplementation decreased *Tlr4* (*p* = 0.026) expression levels (Figure 6b) compared to the HF diet. However, TC and FO did not significantly affect *Mcp1*, *Il1β*, and *Tlr4* expression in B6 female and TH male mice (Figure 6d–i)

In TH female mice, compared to the HF diet, FO supplementation reduced the mRNA expression levels of *Tlr4* (*p* = 0.002) and *Il1β* (*p* = 0.023) (Figure 6k,l), and TC + FO reduced *Tlr4* (*p* = 0.003) expression (Figure 6l).

We also assessed the effects of TC, FO, and the combined TC + FO supplementation on other inflammatory markers, including *Il6*, *Tnfα*, and *Cd11c* genes, as well as anti-inflammatory markers such as *Cd206* and *Ym1*. However, compared to the HF diet, the expression levels of these genes were not significantly altered by any of the supplementations in both B6 and TH (see supplemental figures for details).

### 3.5. Effects of HF Diet on Markers of Fatty Acid Metabolism in WAT

To investigate the effects of the HF diet on lipid metabolism in WAT, we measured the expression level of genes involved in fatty acid synthesis and oxidation.

In B6 mice, two-way ANOVA showed significant main effects of diet on the expression of *Fasn* (F (1, 17) = 16.40, *p* = 0.0008) and *Acaca* (F (1, 18) = 8.29, *p* = 0.01). For *Cpt1*, significant main effects were observed for both sex (F (1, 18) = 24.11, *p* < 0.0001) and diet (F (1, 18) = 14.28, *p* = 0.0014). No interactions between these factors were observed (Table 3).

In TH mice, two-way ANOVA identified significant main effects for *Fasn* and *Acaca*, showing responses to both sex and diet. *Fasn* was influenced by sex (F (1, 20) = 10.26, *p* = 0.0045) and diet (F (1, 20) = 27.04, *p* < 0.0001). *Acaca* also demonstrated significant main effects from sex (F (1, 20) = 18.70, *p* = 0.0003) and diet (F (1, 20) = 34.59, *p* < 0.0001). Also, for *Cpt1* a significant main effect from diet was observed (F (1, 19) = 8.978, *p* = 0.0074). Furthermore, significant interactions between diet and sex were noted for *Fasn* (F (1, 20) = 6.860, *p* = 0.0164) and *Acaca* (F (1, 20) = 11.01, *p* = 0.0034) (Table 4).

**Figure 7:**
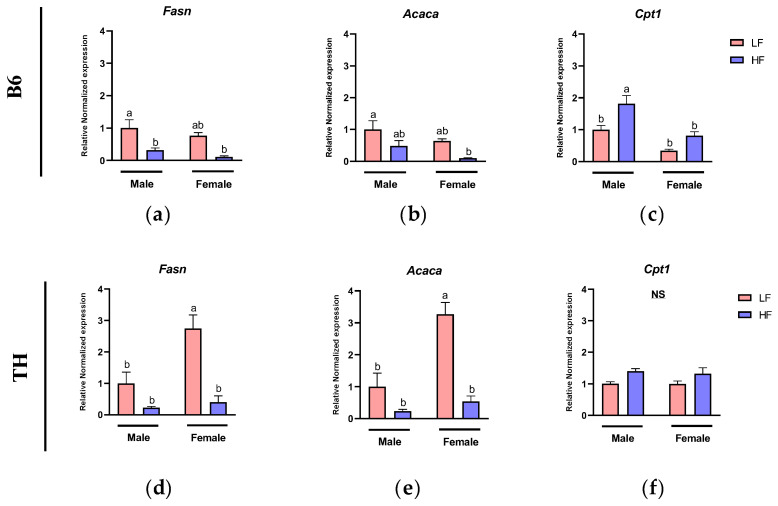
Expression of fat metabolism genes in white adipose tissue (WAT). Fat metabolism markers include (**a**,**d**) Fatty Acid Synthase (*Fasn*), (**b**,**e**) Acetyl-CoA Carboxylase Alpha (*Acaca*), and (**c**,**f**) Carnitine Palmitoyl transferase 1 (*Cpt1*). Data are expressed as mean ± SEM. LF, low fat, HF, high fat. Different letters indicate significant differences between groups. *p* < 0.05; *n* = 6 mice per group.

In B6 male mice, *Fasn* expression levels were significantly lower in the HF group compared to the LF group (*p* = 0.0349) (Figure 7a), while no significant changes were observed in *Acaca* expression levels (Figure 7b). A similar trend was observed in females, with expressions of both *Fasn* and *Acaca* tending to be lower in the HF group compared to the LF one, although no significant difference was observed (Figure 7a,b). In TH mice, when compared to the LF group, the HF group had a lower expression of *Fasn* and *Acaca* mRNA levels in females (*p* < 0.0001) with a similar non-significant trend in males (Figure 7d,e). Regarding fatty acid oxidation, *Cpt1* expression was lower in B6 male mice on the HF diet compared to ones on the LF diet (*p* = 0.0131) (Figure 7c), but no changes were observed in B6 female mice or TH male and female mice (Figure 7c,f).

### 3.6. Effects of Supplemental TC, FO, and Their Combination on Markers of Fatty Acid Metabolism in WAT

To further investigate the effects of TC, FO, and their combination on lipid metabolism in WAT, we analyzed the expression of fat metabolism-related genes, including those related to lipid synthesis and lipid oxidation. In WAT, supplementation of the HF diet with TC, FO, or a combination of both did not modify the expression levels of *Acaca*, *Fasn*, and *Cpt1* compared to the HF diet in both B6 and TH mice (see supplemental figures for details).

### 3.7. Effects of HF Diet on ER Stress Markers in WAT

To investigate the association between obesity-induced inflammation and ER stress, we measured the mRNA levels of ER stress markers, including Total X-box Binding Protein 1 (*TXbp1*), Binding Immunoglobulin Protein (*Bip*), C/EBP Homologous Protein (*Chop*), and Activating Transcription Factor 4 (*Atf4*) in WAT of mice subjected to LF or HF diets for 14 weeks.

In B6 mice, a significant main effect of diet was identified on the expression of *TXbp1* (F (1, 17) = 6.493, *p* = 0.0208) and *Atf4* (F (1, 18) = 10.18, *p* = 0.0051) through two-way ANOVA analysis. Additionally, *Atf4* expression also showed a significant main effect of sex (F (1, 18) = 4.726, *p* = 0.0433). Notably, interactions between diet and sex significantly affected *TXbp1* (F (1, 17) = 7.128, *p* = 0.0162) and *Bip* (F (1, 16) = 9.891, *p* = 0.0063) (Table 3).

In TH mice, significant main effects of sex and diet were identified for *TXbp1*(F (1, 20) = 18.01, *p* = 0.0004, F (1, 20) = 27.74, *p* < 0.0001) and *Chop* (F (1, 20) = 10.80, *p* = 0.0037, F (1, 20) = 25.67, *p* < 0.0001). Also, *Bip* showed a significant main effect just of diet (F (1, 20) = 11.14, *p* = 0.0033). Additionally, the interaction of sex and diet was significant on all ER stress markers including *TXbp1* (F (1, 20) = 4.418, *p* = 0.0484), *Bip* (F (1, 20) = 9.862, *p* = 0.0052), and *Chop* (F (1, 20) = 13.53, *p* = 0.0015) and *Atf4* (F (1, 17) = 13.87, *p* = 0.0017) (Table 4).

**Figure 8:**
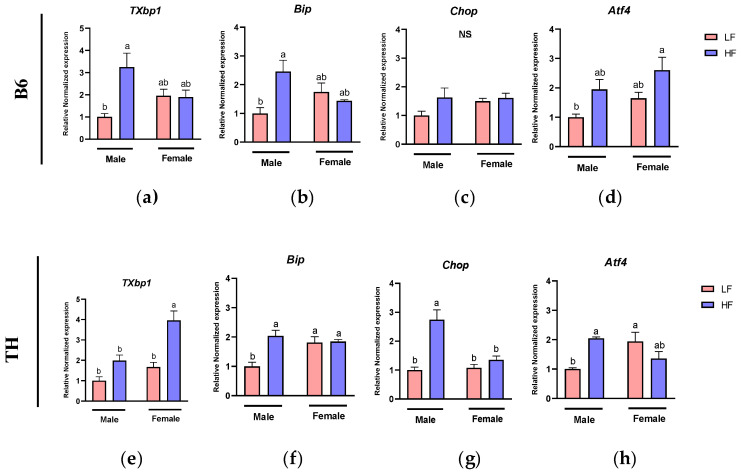
Expression of ER stress-related genes in white adipose tissue (WAT). ER stress markers include (**a**,**e**) Total X-box-binding protein-1 (*TXbp1*), (**b**,**f**) Binding Immunoglobulin Protein (*Bip*), (**c**,**g**) C/EBP Homologous Protein (*Chop*) and (**d**,**h**) Activating Transcription Factor 4 (*Atf4*). Data are expressed as mean ± SEM. LF, low fat, HF, high fat. Different letters indicate significant differences between groups. *p* < 0.05; *n* = 6 mice per group.

In B6 male mice, the expression levels of *TXbp1* (*p* = 0.0069) and *Bip* (*p* = 0.01) were significantly higher in those fed the HF diet compared to ones fed the LF diet (Figure 8a,b). A similar trend was observed for *Chop* and *Atf4*, but no significant differences were detected (Figure 8c,d). However, in B6 female mice, the expression of the above genes was not affected by the HF diet, when compared to the LF diet (Figure 8a–d). In TH male mice, the HF diet resulted in increased expression levels of *Bip* (*p* = 0.0011), *Chop* (*p* < 0.0001), and *Atf4* (*p* = 0.0238) compared to the LF diet (Figure 8f–h). Conversely, in TH female mice, while the HF diet led to elevated *TXbp1* (*p* = 0.0003) expression compared to the LF one (Figure 8e), it did not significantly alter the expression levels of *Bip, Chop*, and *Atf4* (Figure 8f–h).

### 3.8. Effects of Supplemental TC, FO, and Their Combination on ER Stress Markers in WAT

To explore the beneficial effects of TC, FO, and their combination on ER stress in WAT, we next analyzed the gene expression of essential ER stress markers including *Bip*, *Chop*, and *TXbp1* in B6 and TH male and female mice who were fed an HF diet supplemented either with TC, FO, or both.

In B6 mice, two-way ANOVA showed significant main effects of sex on the expression of *Bip* (F (1, 34) = 11.76, *p* = 0.0016) and *Chop* (F (1, 36) = 11.77, *p* = 0.0015). Significant interactions between diet and sex were also detected for *Bip* (F (3, 34) = 9.110, *p* = 0.0001) and *TXbp1* (F (3, 34) = 2.969, *p* = 0.0455), suggesting that the response to diet is modulated by sex in these particular genes (Table 5).

In TH mice, two-way ANOVA demonstrated significant main effects of sex and diet on the expression of *Chop* (sex: F (1, 38) = 11.98, *p* = 0.0013; diet: F (3, 38) = 10.65, *p* < 0.0001) and *TXbp1* (sex: F (1, 37) = 33.99, *p* < 0.0001; diet: F (3, 37) = 7.985, *p* = 0.0003). There was also a significant interaction between diet and sex for *Chop* (F (3, 38) = 6.515, *p* = 0.0012) (Table 6).

**Figure 9:**
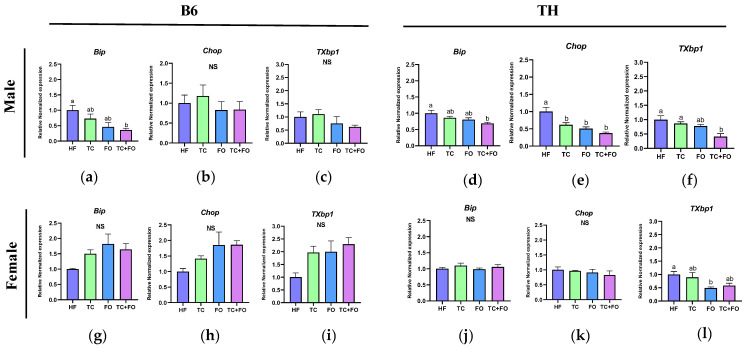
Gene expression levels of (**a**,**d**,**g**,**j**) *Bip*, (**b**,**e**,**h**,**k**) *Chop*, (**c**,**f**,**i**,**l**) *TXbp1* in WAT of mice fed high fat (HF) diets and HF diets supplemented with tart cherry (TC), fish oil (FO), and their combination (TC + FO). Different letters indicate significant differences between groups.

In B6 male mice, the combination of TC and FO significantly decreased the expression level of *Bip* compared to the HF diet (*p* = 0.0321) (Figure 9a). However, when compared to the HF diet, neither TC nor FO, whether administered individually or in combination, affected the expression of *Chop* and *TXbp1* in these mice (Figure 9b,c). Similarly, in B6 female mice, no changes were observed in the expression levels of *Bip*, *Chop*, and *TXbp1* with these supplements (Figure 9g–i).

In TH male mice, the combined TC and FO supplementation significantly downregulated the mRNA levels of *Bip* (*p* = 0.0338), *Chop* (*p* < 0.0001), and *TXbp1* (*p* = 0.002) compared to the HF diet (Figure 9d–f). Additionally, individual supplementation with TC or FO reduced the expression of *Chop* compared to the HF diet (*p* = 0.093 and *p* = 0.0009, respectively) (Figure 9e).

In TH female mice, FO supplementation resulted in a reduction in *TXbp1* expression level compared to the HF group (*p* = 0.0321) (Figure 9l). However, no significant changes were found in the expression levels of *Bip* and *Chop* after any treatment compared to the HF diet (Figure 9j,k).

### 3.9. Effects of HF Diet on Autophagy Markers in WAT

Overaccumulation of fat in adipose tissue disrupts ER functions in adipocytes, causing ER stress and it is associated with dysfunction in autophagy and inflammation. To explore this, we assessed mRNA levels of autophagy markers including Autophagy Related Gene 5 (*Atg5*), Autophagy Related Gene 12 (*Atg12*), and *Beclin1* in WAT of mice on LF or HF diets.

In B6 mice, two-way ANOVA showed significant main effects of diet for *Atg5* (F (1, 20) = 7.771, *p* = 0.0114). For *Beclin1*, significant main effects from both sex (F (1, 18) = 88.08, *p* < 0.0001) and diet (F (1, 18) = 316.6, *p* < 0.0001) were observed. Furthermore, an interaction between diet and sex was detected specifically for *Beclin1* (F (1, 18) = 252.2, *p* < 0.0001) (Table 3).

In TH mice, two-way ANOVA revealed significant main effects of sex and diet on the expression of *Atg5* (F (1, 20) = 44.64, *p* < 0.0001; F (1, 20) = 25.39, *p* < 0.0001). For *Atg12*, significant main effects of diet (F (1, 19) = 33.39, *p* < 0.0001) were observed, along with a significant interaction between diet and sex (F (3, 19) = 6.608, *p* = 0.0187). Additionally, *Beclin1* showed a significant main effect of diet (F (1, 20) = 14.65, *p* = 0.0011) and a significant interaction between diet and sex (F (3, 20) = 5.254, *p* = 0.0329), suggesting that the response to diet varies with sex in these autophagy-related genes (Table 4).

**Figure 10:**
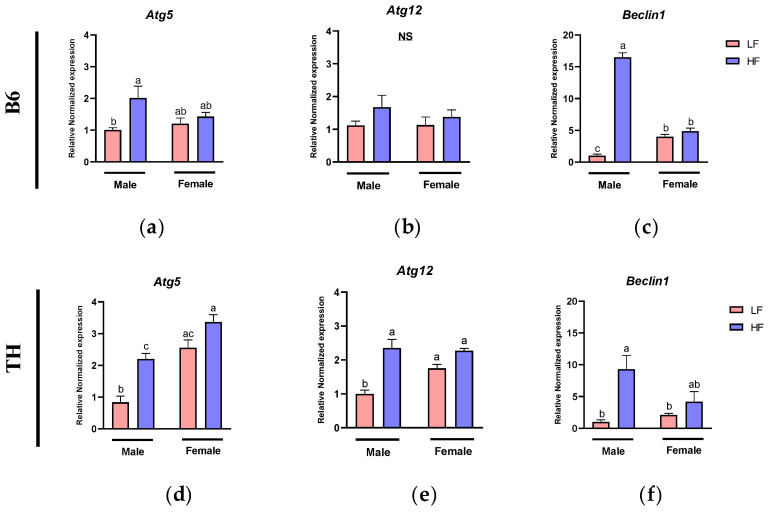
Expression of autophagy-related genes in white adipose tissue (WAT). Autophagy markers include (**a**,**d**) Autophagy Related Gene 5 (*Atg5*), (**b**,**e**) Autophagy Related Gene12 (*Atg12*), and (**c**,**f**) *Beclin1*. Data are expressed as mean ± SEM. LF, low fat, HF, high fat. Different letters indicate significant differences between groups. *p* < 0.05; *n* = 6 mice per group.

In B6 male mice, the mRNA levels of *Atg5* (*p* = 0.02) and *Beclin1* (*p* < 0.0001) were elevated after feeding them the HF diet compared to the LF diet (Figure 10a,c). Also, a similar trend was observed for *Atg12*, although no significant difference was observed (Figure 10b). In B6 female mice, compared to the LF diet, the HF diet slightly increased the expression levels of *Atg5*, *Atg12*, and *Beclin1*, although no significant differences were observed (Figure 10a–c). In TH male mice, the HF diet led to an upregulation of *Atg5* (*p* = 0.0013), *Atg12* (*p* < 0.0001), and *Beclin1* (*p* = 0.002) compared to the LF diet (Figure 10d–f). While a similar trend was observed in TH female mice, the changes in these genes were not statistically significant under the same dietary conditions (Figure 10d–f).

### 3.10. Effects of Supplemental TC, FO, and Their Combination on Autophagy Markers in WAT

To further explore the effects of TC, FO, and their combination on autophagy, we then assessed their impact by examining the expression levels of key autophagy markers, namely *Atg5*, *Atg12*, and *Beclin1*.

In B6 mice, two-way ANOVA revealed significant main effects of sex on the expression of *Beclin1* (F (1, 36) = 10.55, *p* = 0.0025) and *Atg12* (F (1, 38) = 4.943, *p* = 0.0322). Additionally, a significant interaction between diet and sex was found for *Beclin1* (F (3, 36) = 48.30, *p* < 0.0001) and *Atg5* (F (3, 36) = 3.020, *p* = 0.0422) (Table 5).

In TH mice, two-way ANOVA showed significant main effects of diet on the expression of *Beclin1* (F (3, 39) = 5.641, *p* = 0.0026) and *Atg12* (F (3, 37) = 7.071, *p* = 0.0007), and both sex and diet on *Atg5* (sex: F (1, 37) = 54.46, *p* < 0.0001; diet: F (3, 37) = 7.852, *p* = 0.0004). There were no significant interactions between diet and sex for these genes (Table 6).

**Figure 11:**
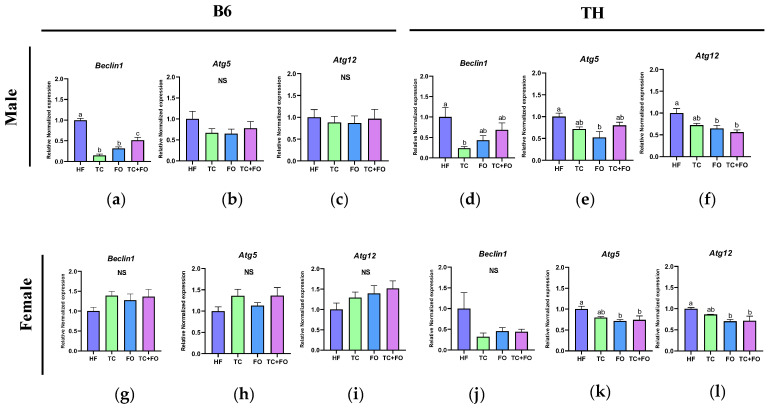
Gene expression levels of (**a**,**d**,**g**,**j**) *Beclin1*, (**b**,**e**,**h**,**k**) *Atg5*, (**c**,**f**,**i**,**l**) *Atg12* in WAT of mice fed high fat (HF) diets and HF diets supplemented with tart cherry (TC), fish oil (FO), and their combination (TC + FO). Group means labeled with different letters are significantly different *p* < 0.05; *n* = 6 mice per group.

In B6 male mice, supplementation of the HF diet with TC, FO, or their combination resulted in a significant reduction in *Beclin1* gene expression compared to the HF group (*p* < 0.0001) (Figure 11a), and the mRNA levels of *Atg5* and *Atg12* were slightly decreased by the treatments (Figure 11b,c). In B6 female mice, no changes were observed in the expression levels of *Beclin1*, *Atg5*, and *Atg12* after any treatment compared to the HF diet (Figure 11g–i).

In TH male mice, TC supplementation reduced the expression level of *Beclin1* compared to the HF diet (*p* = 0.0128) (Figure 11d). In addition, FO supplementation in this group resulted in reduced expression levels of *Atg5* (*p* = 0.0061) and *Atg12* (*p* = 0.0143) compared to the HF diet (Figure 11e,f). Moreover, the combined supplementation of TC and FO significantly lowered the mRNA level of *ATG12* compared to the HF diet in TH male mice (*p* = 0.0021) (Figure 11f).

In TH female mice, both FO and the combination of TC and FO were associated with a decrease in the expression levels of *Atg5* (*p* = 0.0231 for FO, *p* = 0.0461 for TC + FO) and *Atg12* (*p* = 0.0219 for FO, *p* = 0.0284 for TC + FO) when compared to the HF diet (Figure 11k,l).

## 4. Discussion

Previous studies on the beneficial health effects of bioactive compounds have focused on the individual effects of TC and FO in B6 mice, a commonly used model for diet-induced obesity. Our study is the first to investigate potential synergistic or additive effects of TC and FO in both diet-induced and genetically obese mouse models. In our previous paper, we reported that HF diets intensified the obesogenic effect, especially in male TH mice [26]. While neither TC nor the combination of TC + FO improved adiposity or glucose tolerance in either sex of B6 and TH mice, FO alone improved glycemia and glucose tolerance in male TH mice. In the current study, we report that the HF diet increased adipose tissue inflammation, as shown by the upregulation of mRNA expression levels for pro-inflammatory, ER stress, and autophagy markers in both male and female B6 and TH mice.

Both excessive dietary fat intake [29,31] and genetic factors [32] are closely associated with increased body weight and adiposity in various rodent models. We found that genetic predisposition, particularly in male TH mice, significantly accentuated the adiposity response to HF diets. This finding is consistent with a previous study showing that TH mice exhibited significantly higher adiposity on a high-sucrose, high-fat diet compared to a chow diet [25]. Regarding the treatment, we previously reported the effect of high doses of EPA-rich FO (36 g/kg) [33] and purified EPA ethyl ester (36 g/kg) [13,22] in reductions of fat mass. However, a similar effect was not observed in the current study, which may be due to the relatively lower omega-3 PUFA, especially the EPA content. Our observation with TC is consistent with previous studies reporting the anti-inflammatory effect of TC in HF diet-induced obese B6 mice with TC in drinking water [34] and male Zucker obese rats supplemented with 4% TC powder [8], in an adiposity-independent manner. Unlike our expectations, no additive or synergistic effects of TC and FO were observed in alleviating obesity-induced metabolic disorders.

We previously reported that HF diets significantly elevated pro-inflammatory plasma IL-6 levels in all groups of mice, except for B6 males. Both TC and FO supplementation effectively lowered plasma IL-6 levels, with a more substantial reduction observed in females compared to males in both B6 and TH mice [26]. Given the endocrine role of WAT and the recruitment and activation of macrophages in inflamed WAT, which contribute to inflammation and exacerbate metabolic dysregulation [5], this study focused on WAT to further investigate the anti-inflammatory effects of FO and TC. In B6 and TH mice, the expression of *Il6* and *Tlr4* demonstrated a clear main effect of diet, where the HF diet-induced a pro-inflammatory response compared to the LF diet. This underscores the significant role of an HF diet in promoting inflammation, regardless of genetic background. However, the interaction between sex and diet varied between strains. In B6 mice, the HF diet significantly increased *Il6* and *Tnfα*, with notable interactions, suggesting that male B6 mice were more susceptible to HF diet-induced inflammation, possibly due to hormonal influences on immune and metabolic regulation.

Consistent with previous studies [35,36], our results confirmed the pro-inflammatory effect of an HF diet on WAT, as indicated by increased expression levels of pro-inflammatory markers such as *Il6*, *Tnfα*, *Mcp1*, and *Tlr4*. The increased expression of *Il1β* and *Cd80* underscored the shift towards a pro-inflammatory macrophage profile in WAT during obesity. This supports the idea that the rise in M1 macrophages in WAT is closely linked to increased inflammation in obesity [37,38]. A limitation of this study is that we were not able to assess adipocyte size or macrophage infiltration in WAT.

We found that in B6 mice, treatments influenced *Tlr4* in a sex-dependent manner, with males showing a stronger response than females. In TH mice, both *Mcp1* and *Tlr4* exhibited significant main effects of sex and diet, indicating that these inflammatory markers were influenced independently by both factors, with females demonstrating a stronger overall response to the treatments.

Regarding responses to TC and FO supplementation, we observed that TC only slightly reduced *Mcp1* in B6 male mice, while, in our previous study, TC-fed obese male Zucker rats exhibited significant reductions in adipose tissue inflammation, by having reduced mRNA levels of *Mcp1*, *Il6*, *Tnfα*, and *Il1β* [8]. In another study conducted on obese male mice, administration of anthocyanin-rich tart cherry extract (60 mg/kg dissolved in drinking water) significantly reduced plasma levels of leptin and IL-6 [34]. In individuals with overweight and obesity, the consumption of 240 mL of 100% authentic tart cherry juice for 4 weeks reduced plasma Tnfα and Mcp1 without affecting Il6 or Ill0 levels, compared to the placebo group [39]. On the other hand, in our current study, both *Tlr4* and *Il1β* mRNA levels were reduced by FO in WAT of B6 males and TH females, consistent with previously published animal studies reporting FO effects on inflammation in WAT [13,22,40]. In clinical studies, middle-aged and elderly participants with hypertension who were administered a daily dose of 2 g EPA + DHA for 90 days exhibited reduced plasma levels of CRP and Tnfα, while Il6 levels remained unaffected [41]. Regarding the synergistic effect of TC and FO, limited studies have investigated the effects of combined bioactive compounds. Here, supplementation with TC + FO decreased *Mcp1* and *Tlr4* mRNA levels in B6 male mice and *Tlr4* in TH female mice. However, no synergistic or additive effects were observed following supplementation with TC + FO compared to TC and FO administered individually. The absence of synergistic or additive effects could be attributed to several factors. First, the doses of TC and FO used may not have been optimal doses, as previous studies, particularly for FO, have demonstrated more potent anti-inflammatory effects at higher doses [42]. Additionally, the timing and duration of supplementation on a high fat and sucrose diet may not have been sufficient to observe synergy. Thus, the limitation of our study includes a lack of varying supplementation doses and durations with FO and TC to assess individual and interactive effects. Moreover, the complexity of obesity-related pathways such as inflammation, may resist additive or synergistic interactions, and genetic differences between B6 and TH mice could have influenced the metabolism and response to these interventions.

We also investigated the molecular metabolic changes of WAT under HF-diet exposure in both B6 and TH mice, finding that the HF diet reduced the expression of genes involved in lipogenesis while it increased the expression of genes involved in fatty acid oxidation, a common metabolic adaptation in both strains. Additionally, in TH mice, a significant sex-diet interaction for *Fasn* and *Acaca*, indicated that TH female mice exhibited a larger reduction in lipogenic response to the HF diet compared to males, with reduced *Fasn* and *Acaca* levels in WAT. This finding aligns with research by Anja Voigt et al., which showed that mice on semisynthetic diets with 10% (LF) or 40% (HF) energy from fat over 5 days to 12 weeks exhibited reductions in both *Fasn* and *Acaca* mRNA levels on the HF diet compared to the LF one [43]. With regards to fatty acid oxidation, the expression of *Cpt1* was higher in response to HF feeding in both male (significant) and female (trending) B6 mice, possibly indicating an increased fat oxidation in response to higher availability of fat. Interestingly, this HF effect on fatty acid oxidation, as indicated by *Cpt1* expression, was blunted in both TH male and female mice. Whether this lack of adaptive increase in fatty acid oxidation is responsible for excessive adiposity seen in TH mice fed an HF diet warrants further investigation. In line with this, a previous study has reported impaired mitochondrial oxidative phosphorylation in TH mice [44]. Whether the blunted increase in *Cpt1* expression and impaired oxidative phosphorylation in TH mice is due to mitochondrial dysfunction merits further study.

Surprisingly, TC, FO, and their combination in both TH and B6 mice did not alter the expression levels of genes related to fatty acid metabolism in WAT when compared to those on the HF diet; this could be attributed to several factors. First, the HF diet itself may have already downregulated lipogenic pathways to a point where the addition of these bioactive compounds could not further affect these metabolic processes. Second, the doses of TC and FO used may not have been sufficient to elicit further reductions in fat metabolizing genes. Additionally, fat metabolism is a tightly regulated process, influenced by genetic and environmental factors, which might limit the potential for dietary interventions like TC and FO to modulate these pathways significantly.

To investigate the link between obesity-induced inflammation and ER stress, we measured mRNA levels of ER stress markers in WAT of HF-fed obese mice. We found that both B6 and TH mice showed significant main effects of diet on ER stress markers, indicating increased ER stress under HF diet exposure. In TH mice, there was a significant interaction between sex and diet across all ER stress markers, and male mice exhibited a more pronounced ER stress response compared to females. We observed upregulation of *TXbp1* and *Bip* in B6 male mice, with TH male mice also showing increased levels of *Bip*, *Chop*, and *Atf4* due to the HF diet. These findings are consistent with a similar study where a 16-week HF diet led to heightened expressions of both inflammatory and ER stress markers (*Tnfα*, *Il1β*, *Il6*, *Mcp1*, *Bip*, and *Chop/Atf4*) in gonadal adipose tissue of B6 male mice [45]. Suzuki et al. showed that CHOP upregulation in adipocytes of HFD-fed mice exacerbates adipose tissue inflammation by promoting Th2 cytokine production and M1 macrophage recruitment. Further, Chop deficiency led to reduced macrophage infiltration, highlighting the role of Chop in mediating ER stress-induced adipose inflammation [46]. Overall, our data indicate that prolonged obesity induces inflammation and ER stress and activates the unfolded protein response (UPR) signaling pathway in adipocytes, underscoring the intricate relationship between obesity-induced inflammation and ER stress mechanisms.

In TH mice, there were significant main effects of sex and diet for both *Chop* and *TXbp1*, indicating that both factors independently influenced ER stress levels. Additionally, in TH mice there was a significant interaction between sex and diet for *Chop*, suggesting that male mice exhibited a stronger ER stress response to the HF diet compared to females.

The effects of TC and omega-3 PUFAs on ER stress, individually, have been reported by previous studies. For example, a study involving diet-induced obese (DIO) male rats demonstrated that supplements of TC seed powder and a combination of the seed powder with juice effectively elevated GRP94 protein levels, thus improving ER efficiency [47]. Another study involving male Wistar rats fed an HF diet observed that administering blueberry juice rich in anthocyanins over 8 weeks led to a reduction in the expression of ER stress markers IRE-1 and Chop, indicating suppression of the IRE-1 and PERK signaling pathways [48]. Regarding FO, a study demonstrated that FO supplementation significantly reduced phosphorylated eIF2α and Chop in B6 male mice on an HF diet. This was evidenced by both the reduced protein expression of these markers and the down-regulation of related mRNA expression [49]. In the current study, we investigated their combined effects and found that TC supplementation significantly reduced *Chop* and *TXbp1* expression in TH male mice, and combining TC with FO further decreased key ER stress markers (*Bip*, *Chop*, and *TXbp1*) in TH male WAT.

Lastly, we accessed the autophagy level, a critical lysosomal degradation pathway that plays an important role in maintaining cellular homeostasis by recycling damaged molecules and organelles [50,51] in WAT. Beyond its integral role in the differentiation of adipocytes, autophagy is essential for promoting adipose tissue hypertrophy and regulating lipid storage [52]. Dysfunctional autophagy is linked to abnormal adipose tissue expansion and subsequent development of chronic diseases [53,54]. We found that there was a significant main effect of diet on autophagy markers in both B6 and TH mice, indicating increased autophagic activity in response to diet-induced stress. In TH mice, significant interactions between sex and diet for *Atg12* and *Beclin1* revealed that TH males showed a stronger response compared to females.

Here, we demonstrated an increase in the mRNA levels of *Atg5*, *Atg12*, and *Beclin1* in the WAT of both B6 and TH male mice, indicating that an HF diet promotes the initiation of autophagy, particularly the formation of autophagosomes. This is consistent with previous research showing elevated mRNA levels of *Atg5* and *Atg7*, as well as higher protein levels of Lc3II in the WAT of HF diet-induced obese mice [55]. Similar, Yovita et al., demonstrated a significant increase in the expression of Lc3-II and Atg5 after 16 weeks of an HF diet in male mice [56]. Furthermore, the existing literature confirms that HF diet-induced obesity enhances autophagosome formation [57,58,59]. However, there are contrasting findings, such as Zhang et al., who noted a decrease in the LC3-II/LC3-I ratio, reflecting diminished autophagosome formation in HFD-induced obesity [60], and Sakane et al., who observed no changes in autophagosome formation markers like Atg5, Lc3, and Beclin1in HF diet-fed mice [61]. The discrepancies among the above findings could stem from variations in the HF diet composition and the experimental methodologies applied across studies. In B6 mice, there was a significant main effect of sex and diet for *Beclin1*, with a notable interaction between sex and diet, indicating that males showed a more pronounced autophagic response to the HF diet compared to females. In TH mice, both *Beclin1* and *Atg5* showed significant main effects of sex and diet. However, there were no significant sex and diet interactions in TH mice, suggesting a more consistent response across sexes to the diet.

Regarding the treatment, we observed that supplementation with FO and a combination of TC and FO downregulated *Atg5* and *Atg12* gene expression compared to the HF diet in both TH male and female mice but not in B6 mice. Similarly, fat-1 transgenic mice, which overproduce endogenous omega-3 PUFAs, were first treated with t-TUCB, a soluble epoxide hydrolase (sEH) inhibitor that prevents the breakdown of beneficial omega-3-derived epoxides. This pre-treatment was followed by a high-fat diet, leading to increased omega-3-derived CYP epoxides, modified protein levels, reduced LC3-II, and elevated P62 in epididymal WAT, indicating a decrease in autophagic flux [62]. Further supporting our findings, a study involving jaboticaba peel extract (FJE), rich in anthocyanins, showed that FJE treatment decreases the protein levels of Beclin-1 and LC3BII and increases the protein level of SQSTM-p62 in the epididymal WAT of mice fed an HF diet, illustrating the impact of anthocyanins on autophagy modulation in obesity contexts [63].

While the current study focused on the effects of FO, TC, and their combination in adipose tissue, it is plausible that the effects of these compounds on other tissues such as the liver and skeletal muscle are also important in their overall whole body metabolic effects. In this regard, we did not find marked effects of these interventions in the livers of these mice (Supplementary Figures).

## 5. Conclusions

In conclusion, our study provides insights into the individual and combined effects of TC and FO on inflammation in diet-induced and genetically obese mice. While individually, TC and FO showed clear anti-inflammatory effects, particularly in modulating adipose tissue inflammation, we did not observe any major consistent synergistic or additive effects. This may be attributed to dosage levels, duration of intervention, and the complexity of obesity-related metabolic pathways given the high caloric high fat and high sucrose diets used. Additionally, the genetic differences between B6 and TH mice likely influenced the response to these bioactive compounds. Our findings highlight the intricate role of genetic predisposition in obesity and suggest that while TC and FO hold the potential for reducing obesity-related inflammation, further studies are required to determine the optimal concentrations of these bioactive compounds to maximize their beneficial effects and ensure more pronounced therapeutic outcomes. Taken together, this study highlights the heterogenic responses of both high-fat diet and dietary bioactives on different genetic backgrounds and sex, underscoring the importance of personalized nutrition approaches.

Based on our previously reported stronger effects of FO when we used EPA-enriched FO, the smaller effects observed here may be due to the low levels of EPA in the Menhaden oil used. Thus, future studies comparing EPA to DHA and how each affects responses to TC are worthwhile investigating. These findings also support the continued investigation into the modulation of inflammation, ER stress, and autophagy as potential mechanisms for dietary interventions in obesity management.

## Figures and Tables

**Table 1:** Diet composition.

	LF	HF	HF	HF	HF
			TC	FO	TC + FO
Ingredient (g)					
Casein	200	200	195.6	200	195.6
Corn starch	427	38	35.5	38	35.5
Sucrose	172.8	172.8	144	172.8	144
Soybean oil	25	25	25	25	25
Lard	20	193	193	144	144
Menhaden oil	0	0	0	49	49
Tart cherry powder	0	0	43	0	43
Total	1055	849	855	849	855
g%					
Protein	17.0	21.1	20.9	21.1	20.9
Carbohydrate	66.3	37.8	37.5	37.8	37.5
Fat	4.5	26.0	25.8	26.0	25.8
Fiber	4.7	5.9	6.3	5.9	6.3
Tart cherry powder	0.0	0.0	5.0	0.0	5.0
Kcal%					
Protein	18	18	18	18	18
Carbohydrate	71	32	32	32	32
Fat	11	50	50	50	50

LF, low fat diets; HF, high fat diets; TC, tart cherry; FO, fish oil.

**Table 2:** Primer list and sequences.

Gene	Forward	Reverse
*Il6*	AACCGCTATGAAGTTCCTCTC	TCCTCTGTGAAGTCTCCTCTC
*Tnfa*	CGTGGAACTGGCAGAAGAG	TGAGAAGAGGCTGAGACATAGG
*Mcp1*	ACTTCTATGCCTCCTGCTCAT	GCTGCTTGTGATTCTCCTGTAG
*Tlr4*	AGTAGCACTGACACCTTCCTT	GCCTTAGCCTCTTCTCCTTCA
*Il1β*	GTCACAAGAAACCATGGCACAT	GCCCATCAGAGGCAAGGA
*Cd80*	GTCGTCGTCATCGTTGTCAT	CCGAAGGTAAGGCTGTTGTT
*Arg1*	CCTATCACCGCAGAACCT	GCATCATACAACGAGGAGTG
*Fasn*	TGCAGAAGATGTAGATTGTGTGATGA	GGGTCCGGGTGCAGTTTATT
*Acaca*	GCAGCAGTTACACCACATACA	CATTACCTCAATCTCAGCATAGCA
*Cpt1a*	GAGACAGACACCATCCAACAC	GAGCCAGACCTTGAAGTAACG
*TXbp1*	TGGCCGGGTCTGCTGAGTCCG	GTCCATGGGAAGATGTTCTGG
*Bip*	TTCAGCCAATTATCAGCAAACTCT	TTTTCTGATGTATCCTCTTCACCAGT
*Chop*	CCACCACACCTGAAAGCAGAA	AGGTGAAAGGCAGGGACTCA
*Atf4*	GGGTTCTGTCTTCCACTCCA	AAGCAGCAGAGTCAGGCTTTC
*Atg5*	TCAGAAGGTTATGAGACAAGAAGA	GGATGGACAGTGTAGAAGGT
*Atg12*	AGCAGGAAGAGTGAACCA	AAGCACATAGAGACGAGAAGT
*Beclin1*	GAGATTGGACCAGGAGGAA	AGGTGGCATTGAAGACATTG

**Table 3:** Two-way ANOVA. Effects of sex and diet (LF vs. HF) on gene expression of inflammation, lipid metabolizing, ER stress, and autophagy markers in B6 male and female mice. *p* and F values of two-way ANOVA to evaluate main effects and interactions.

LF vs. HF		Main Effects		Interactions
Variable	Statistic	Sex (S)	Diet (D)	S × D
*Il6*	*p*	**0.0005**	**0.0141**	**0.0136**
	F (1, 18)	17.59	7.388	7.489
*Tnfα*	*p*	0.1472	**0.0191**	**0.0336**
	F (1, 17)	2.307	6.702	5.341
*Mcp1*	*p*	0.1163	0.0546	0.2293
	F (1, 14)	2.803	4.398	1.580
*Tlr4*	*p*	0.8333	**<0.0001**	0.1185
	F (1, 15)	0.04587	39.04	2.742
*Il1β*	*p*	0.0782	**0.0074**	0.0804
	F (1, 17)	3.512	9.251	3.456
*Cd80*	*p*	**0.0419**	**0.0019**	0.2910
	F (1, 18)	4.796	13.23	1.184
*Arg1*	*p*	**0.0002**	**<0.0001**	**0.0178**
	F (1, 16)	23.74	43.20	6.978
*Fasn*	*p*	0.1998	**0.0008**	0.9311
	F (1, 17)	1.779	16.40	0.007694
*Acaca*	*p*	0.0593	**0.0100**	0.9628
	F (1, 18)	4.054	8.290	0.002242
*Cpt1*	*p*	**0.0001**	**0.0014**	0.3152
	F (1, 18)	24.11	14.28	1.067
*TXbp1*	*p*	0.6466	**0.0208**	**0.0162**
	F (1, 17)	0.2178	6.493	7.128
*Bip*	*p*	0.6358	0.0573	0.0063
	F (1, 16)	0.2331	4.197	9.891
*Chop*	*p*	0.2832	0.1055	0.2537
	F (1, 18)	1.224	2.905	1.390
*Atf4*	*p*	**0.0433**	**0.0051**	0.9859
	F (1, 18)	4.726	10.18	0.0003198
*Atg5*	*p*	0.3899	**0.0114**	0.0896
	F (1, 20)	0.7725	7.771	3.184
*Atg12*	*p*	0.5629	0.1215	0.5454
	F (1, 18)	0.3474	2.641	0.3799
*Beclin1*	*p*	**<0.0001**	**<0.0001**	**<0.0001**
	F (1, 18)	88.08	316.6	252.2

**Table 4:** Two-way ANOVA. Effects of sex and diet (LF vs. HF) on gene expression of inflammation, lipid metabolizing, ER stress, and autophagy markers in TH male and female mice. *p* and F values of two-way ANOVA to evaluate main effect and interactions.

LF vs. HF		Main Effects		Interactions
Variable	Statistic	Sex (S)	Diet (D)	S × D
*Il6*	*p*	**0.0007**	**<0.0001**	0.9186
	F (1, 19)	16.28	59.38	0.01073
*Tnfα*	*p*	0.3128	0.0823	0.1703
	F (1, 18)	1.079	3.386	2.040
*Mcp1*	*p*	**0.0114**	**0.0053**	0.3187
	F (1, 20)	7.770	9.775	1.046
*Tlr4*	*p*	**0.0007**	**0.0094**	0.9304
	F (1, 20)	16.14	8.244	0.007814
*Il1β*	*p*	**<0.0001**	**0.0006**	0.0774
	F (1, 18)	39.10	17.23	3.509
*Cd80*	*p*	0.1935	**<0.0001**	0.6845
	F (1, 20)	1.810	40.65	0.1700
*Arg1*	*p*	**0.0033**	**<0.0001**	0.3014
	F (1, 20)	11.13	35.53	1.125
*Fasn*	*p*	**0.0045**	**<0.0001**	**0.0164**
	F (1, 20)	10.26	27.04	6.860
*Acaca*	*p*	**0.0003**	**<0.0001**	**0.0034**
	F (1, 20)	18.70	34.59	11.01
*Cpt1*	*p*	0.7260	**0.0074**	0.7667
	F (1, 19)	0.1265	8.978	0.09060
*TXbp1*	*p*	**0.0004**	**<0.0001**	**0.0484**
	F (1, 20)	18.01	27.74	4.418
*Bip*	*p*	**0.0660**	**0.0033**	**0.0052**
	F (1, 20)	3.781	11.14	9.862
*Chop*	*p*	**0.0037**	**<0.0001**	**0.0015**
	F (1, 20)	10.80	25.67	13.53
*Atf4*	*p*	0.5831	0.2961	**0.0017**
	F (1, 17)	0.3131	1.162	13.87
*Atg5*	*p*	**<0.0001**	**<0.0001**	0.2165
	F (1, 20)	44.64	25.39	1.629
*Atg12*	*p*	0.0527	**<0.0001**	**0.0187**
	F (1, 19)	4.272	33.39	6.608
*Beclin1*	*p*	0.1516	**0.0011**	**0.0329**
	F (1, 20)	2.223	14.65	5.254

**Table 5:** Two-way ANOVA. Effects of sex and diet (HF vs. treatments) on gene expression of inflammation, lipid metabolizing, ER stress, and autophagy markers in B6 male and female mice. *p* and F values of two-way ANOVA to evaluate main effect and interactions.

HF vs. Treatments		Main Effects		Interactions
Variable	Statistic	Sex (S)	Diet (D)	S × D
*Mcp1*	*p*	0.9336	0.1660	0.0813
	F (1, 30)	0.007051		
	F (3, 30)		1.813	2.467
*Tlr4*	*p*	**<0.0001**	0.1599	**0.0002**
	F (1, 30)	34.97		
	F (3, 30)		1.847	8.887
*Il1β*	*p*	0.2391	0.0527	0.3453
	F (1, 34)	1.436		
	F (3, 34)		2.834	1.144
*Bip*	*p*	**0.0016**	0.9487	**0.0001**
	F (1, 34)	11.76		
	F (3, 34)		0.1183	9.110
*Chop*	*p*	**0.0015**	0.4290	0.0651
	F (1, 36)	11.77		
	F (3, 36)		0.9452	2.627
*TXbp1*	*p*	0.1737	0.3852	**0.0455**
	F (1, 34)	1.931		
	F (3, 34)		1.045	2.969
*Beclin1*	*p*	**0.0025**	**<0.0001**	**<0.0001**
	F (1, 36)	10.55		
	F (3, 36)		30.41	48.30
*Atg5*	*p*	0.2895	0.3579	**0.0422**
	F (1, 36)	1.156		
	F (3, 36)		1.110	3.020
*Atg12*	*p*	**0.0322**	0.2548	0.1505
	F (1, 38)	4.943		
	F (3, 38)		1.410	1.873

**Table 6:** Two-way ANOVA. Effects of sex and diet (HF vs. treatments) on gene expression of inflammation, lipid metabolizing, ER stress, and autophagy markers in TH male and female mice. *p* and F values of two-way ANOVA to evaluate main effect and interactions.

HF vs. Treatments		Main Effects		Interactions
Variable	Statistic	Sex (S)	Diet (D)	S × D
*Mcp1*	*p*	**0.0019**	**0.0430**	0.4679
	F (1, 38)	11.11		
	F (3, 38)		2.988	0.8644
*Tlr4*	*p*	**0.0088**	**0.0068**	0.1877
	F (1, 38)	7.640		
	F (3, 38)		4.721	1.679
*Il1β*	*p*	**0.0003**	0.0891	**0.0454**
	F (1, 35)	15.76		
	F (3, 35)		2.351	2.962
*Bip*	*p*	0.4521	0.2923	0.3137
	F (1, 39)	0.5769		
	F (3, 39)		1.287	1.225
*Chop*	*p*	**0.0013**	**<0.0001**	**0.0012**
	F (1, 38)	11.98		
	F (3, 38)		10.65	6.515
*TXbp1*	ps	**<0.0001**	**0.0003**	0.1131
	F (1, 37)	33.99		
	F (3, 37)		7.985	2.129
*Beclin1*	*p*	0.1099	**0.0026**	0.5592
	F (1, 39)	2.677		
	F (3, 39)		5.641	0.6975
*Atg5*	*p*	**<0.0001**	**0.0004**	0.6903
	F (1, 37)	54.46		
	F (3, 37)		7.852	0.4916
*Atg12*	*p*	0.0613	**0.0007**	0.4191
	F (1, 37)	3.725		
	F (3, 37)		7.071	0.9658

## Data Availability

Data supporting reported results can be requested after publication from the corresponding author.

## Supplementary Materials

The following supporting information can be downloaded at: https://www.mdpi.com/article/10.3390/nu16234179/s1, Figure S1. Gene expression levels of (a,d,g,j) *Il6*, (b,e,h,k) *Tnfα*, (c,f,i,l) *Tlr2* in WAT of mice fed high fat (HF) diets and HF diets supplemented with tart cherry (TC), fish oil (FO), and their combination (TC + FO). Group means labeled with different letters are significantly different *p* < 0.05; n = 6 mice per group. Figure S2. Gene expression levels of (a,d,g,j) *Acaca*, (b,e,h,k) *Fasn*, (c,f,i,l) *Cpt1* in WAT of mice fed high fat (HF) diets and HF diets supplemented with tart cherry (TC), fish oil (FO), and their combination (TC + FO). Group means labeled with different letters are significantly different *p* < 0.05; *n* = 6 mice per group. Figure S3. Expression of inflammation-related genes in liver tissue. Liver inflammatory marker genes include (a,c) Interleukin b (*Ilb*), (b,d) Toll-like receptor 4 (*Tlr4*), Data are expressed as mean ± SEM. LF, low fat, HF, high fat. *p* < 0.05; *n* = 6 mice per group. Figure S4. Gene expression levels of (a,c,e,g) *Ilb*, (b,d,f,h) *Tlr4* in liver tissue of mice fed high fat (HF) diets and HF diets supplemented with tart cherry (TC), fish oil (FO), and their combination (TC + FO). Group means labeled with different letters are significantly different *p* < 0.05; *n* = 6 mice per group. Figure S5. Expression of fat metabolism genes in liver tissue. Fat metabolism markers include (a,d) Fatty Acid Synthase (*Fasn*), (b,e) Acetyl-CoA Carboxylase Alpha (*Acaca*), and (c,f) Carnitine Palmitoyl transferase 1 (*Cpt1*). Data are expressed as mean ± SEM. LF, low fat, HF, high fat. *p* < 0.05; *n* = 6 mice per group. Figure S6. Gene expression levels of (a,d,g,j) *Acaca*, (b,e,h,k) *Fasn*, (c,f,i,l) *Cpt1* in liver tissue of mice fed high fat (HF) diets and HF diets supplemented with tart cherry (TC), fish oil (FO), and their combination (TC + FO). Group means labeled with different letters are significantly different *p* < 0.05; *n* = 6 mice per group.
