# Supplementary material for: Mechanisms Mediating Tart Cherry and Fish Oil Metabolic Effects in Diet-Induced (C57BL/6J) and Genetically (TALYHO/Jng) Obese Mice
Source: Nutrients. 2024 Dec 1;16(23):4179. doi: 10.3390/nu16234179 (PMC11644550; doi:10.3390/nu16234179)
Supplement: Supplementary file 1 [file nutrients-16-04179-s001.zip › nutrients-3303154-supplementary.pdf]

Supplemental figures

Article

Mechanisms mediating tart cherry and fish oil metabolic effects in diet-induced (C57BL/6J) and genetically (TALYHO/Jng) obese mice

Maryam Seifishahpar[1,2], Jung Han Kim[3], Jacaline K. Parkman[3], Ana Rhode[1,2], Kalhara Menikdiwela[1,2], Yujiao Zu[1,2], Shane Scoggin[1,2], Logan Freeman [3], Nishan Sudheera Kalupahana[4], Naima Moustaid-Moussa[1,2,]

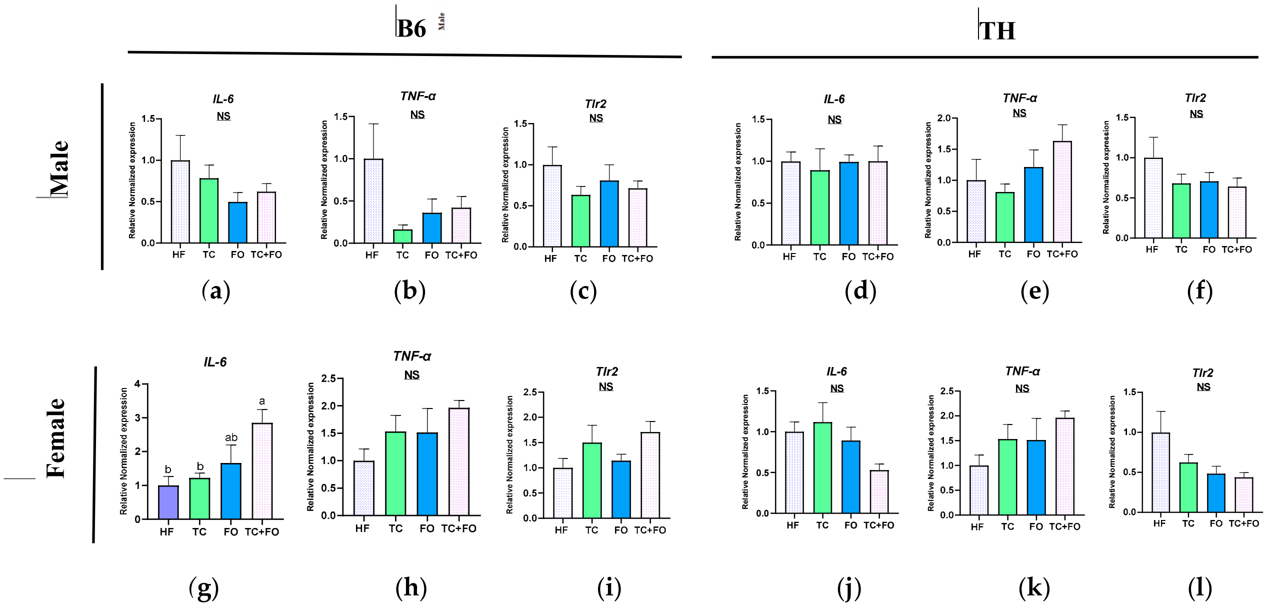

Figure S1. Gene expression levels of (**a, d, g, j**) *Il6*, (**b, e, h, k**) *Tnfα*, (**c, f, i, l**) *Tlr2* in WAT of mice fed high fat (HF) diets and HF diets supplemented with tart cherry (TC), fish oil (FO), and their combination (TC+FO). Group means labeled with different letters are significantly different $P < 0.05$; $n = 6$ mice per group.

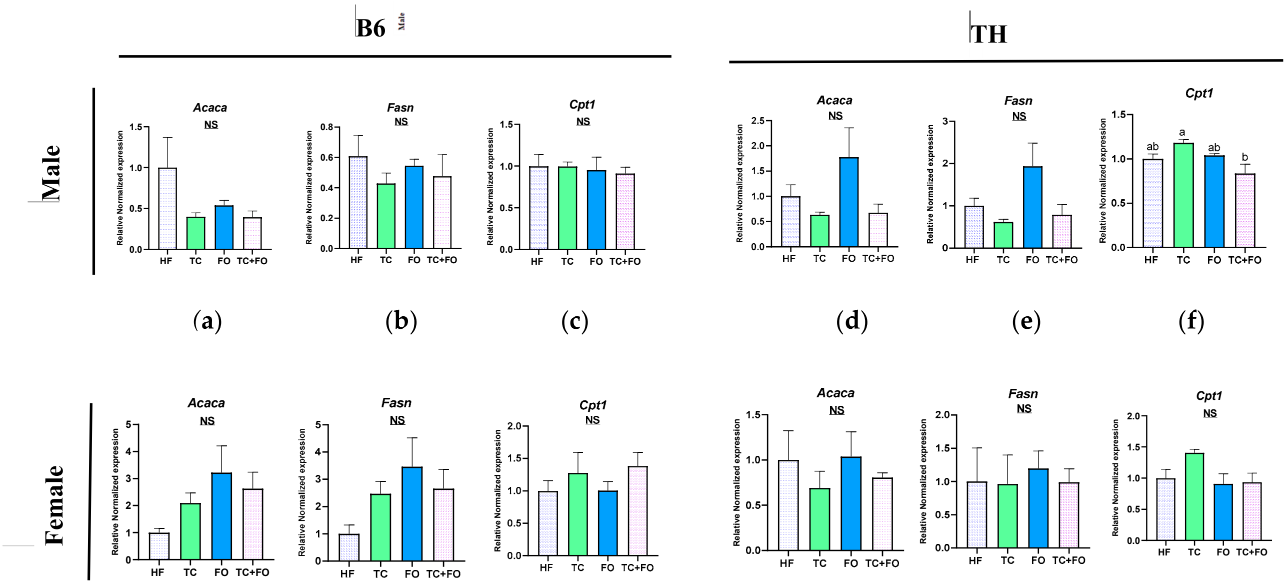

(a) (b) (c) (d) (e) (f)

(g) (h) (i) (j) (k) (l)

Figure S2. Gene expression levels of (**a, d, g, j**) *Acaca*, (**b, e, h, k**) *Fasn*, (**c, f, i, l**) *Cpt1* in WAT of mice fed high fat (HF) diets and HF diets supplemented with tart cherry (TC), fish oil (FO), and their combination (TC+FO). Group means labeled with different letters are significantly different P< 0.05; n = 6 mice per group.

Liver Gene Expression Data

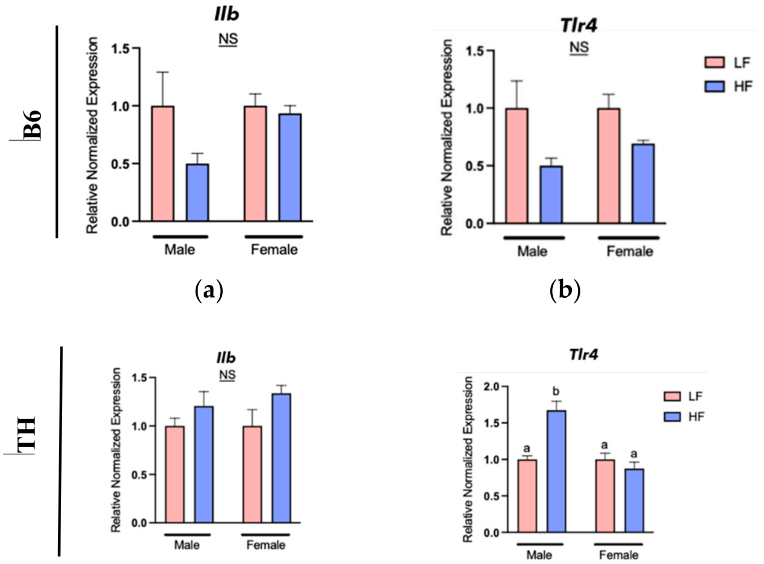

(a) (b)

(c) (d)

Figure S3. Expression of inflammation-related genes in liver tissue. Liver inflammatory marker genes include (**a, c**) Interleukin b (*Ilb*), (**b, d**) Toll-like receptor 4 (*Tlr4*), Data are expressed as mean ± SEM. LF, low fat, HF, high fat. P < 0.05; *n* = 6 mice per group.

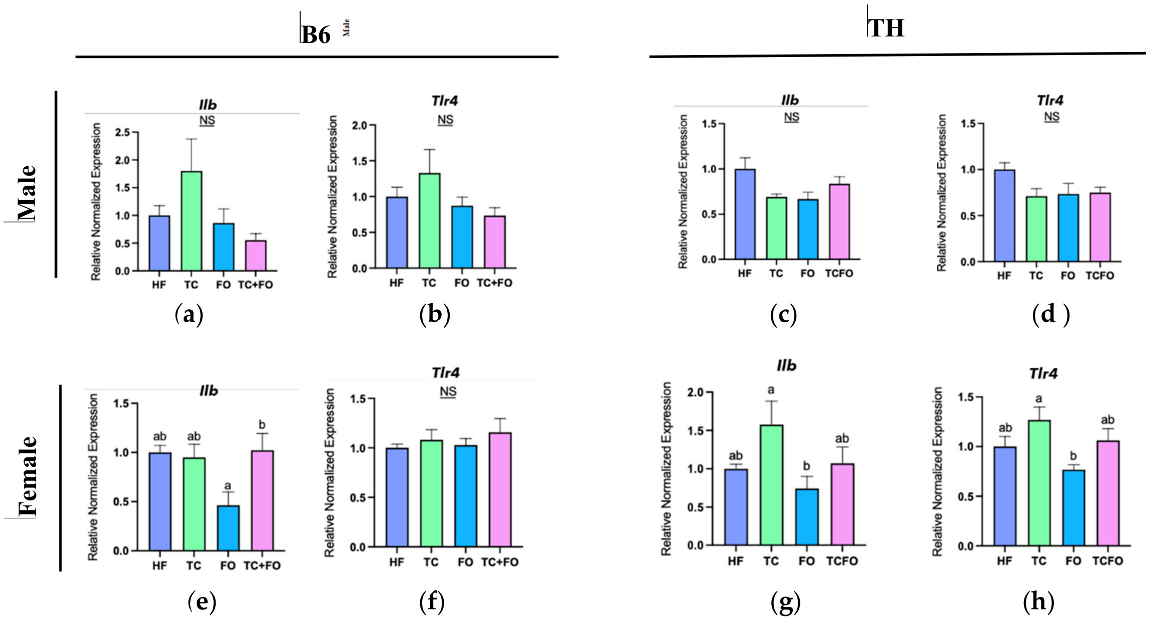

Figure S4. Gene expression levels of (**a**, **c**, **e**, **g**) *Ilb*, (**b, d, f, h**) *Tlr4* in liver tissue of mice fed high fat (HF) diets and HF diets supplemented with tart cherry (TC), fish oil (FO), and their combination (TC+FO). Group means labeled with different letters are significantly different P< 0.05; *n* = 6 mice per group**.**

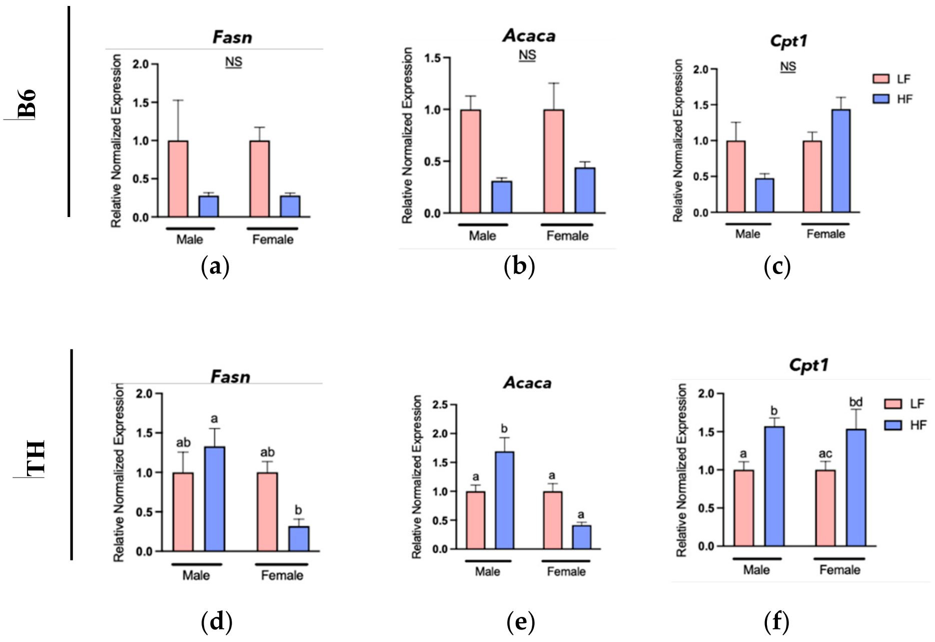

Figure S5. Expression of fat metabolism genes in liver tissue. Fat metabolism markers include (**a**, **d**) Fatty Acid Synthase (*Fasn*), (**b, e**) Acetyl-CoA Carboxylase Alpha (*Acaca*), and (**c, f**) Carnitine Palmitoyl transferase 1 (*Cpt1*). Data are expressed as mean ± SEM. LF, low fat, HF, high fat. $p < 0.05$; $n = 6$ mice per group.

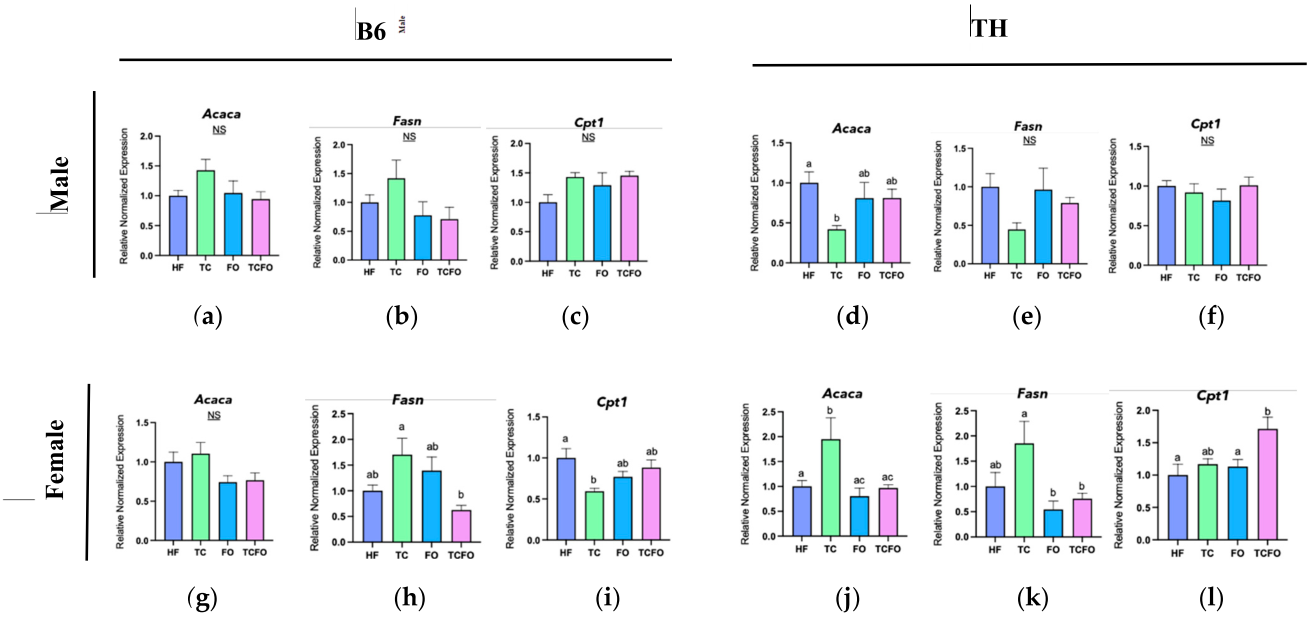

Figure S6. Gene expression levels of (**a, d, g, j**) *Acaca*, (**b, e, h, k**) *Fasn*, (**c, f, i, l**) *Cpt1* in liver tissue of mice fed high fat (HF) diets and HF diets supplemented with tart cherry (TC), fish oil (FO), and their combination (TC+FO). Group means labeled with different letters are significantly different P< 0.05; n = 6 mice per group.
